# Risk factors for progression toward brain death after out-of-hospital cardiac arrest

**DOI:** 10.1186/s13613-019-0520-0

**Published:** 2019-04-08

**Authors:** Martin Cour, Jean Turc, Thomas Madelaine, Laurent Argaud

**Affiliations:** 1 Hospices Civils de Lyon, Hôpital Edouard Herriot, Service de Médecine Intensive-Réanimation, 5, Place d’Arsonval, 69437 Lyon Cedex 03, France; 2 Faculté de médecine Lyon-Est, Université Claude Bernard Lyon 1, Université de Lyon, 69373 Lyon, France; 3 U1060 CarMeN, INSERM, 69373 Lyon, France

**Keywords:** Cardiac arrest, Resuscitation, Brain death, Organ donation, Prognostication, Post-cardiac arrest syndrome

## Abstract

**Background:**

Successfully resuscitated out-of-hospital cardiac arrest (OHCA) may lead to brain death (BD) and good-quality transplantable organs. We aimed to determine risk factors for evolution toward BD after OHCA. We analyzed adult patients admitted to an intensive care unit (ICU) who survived at least 24 h after an OHCA between 2005 and 2015. BD was defined according to international guidelines. Multivariate logistic regression was used to identify potential risk factors for BD available 24 h after OHCA.

**Results:**

A total of 214 patients were included (median age 68 years; sex ratio 1.25; non-shockable OHCA: 88%). Among these, 42 (19.6%) developed BD, while 22 (10.3%) were alive at 1 year with a good neurological outcome. Independent risk factors for BD were age (OR per year 0.95; 95% CI [0.92–0.98]), female gender (OR 2.34; 95% CI [1.02–5.35]), neurological cause of OHCA (OR 14.72; 95% CI [3.03–71.37]), duration of the low-flow period > 16 min (OR 2.94, 95% CI [1.21–7.16]) and need of vasoactive drugs at 24 h (OR 6.20, 95% CI [2.41–15.93]).

**Conclusions:**

The study identified, in a population of OHCA with predominantly non-shockable initial rhythms, five simple risk factors independently associated with progression toward BD.

## Background

Each year more than 300,000 patients experience an out-of-hospital cardiac arrest (OHCA) in both Europe and the USA [1, 2]. Even in patients in whom successful return of spontaneous circulation (ROSC) has been obtained, prognosis remains very poor [2]. This is particularly true for the increasing number of patients resuscitated by the emergency medical system presenting with non-shockable cardiac arrest rhythms (i.e., pulseless electrical activity and asystole), now accounting for approximately three-quarters of OHCAs [1, 2]. Overall, < 10% of the patients admitted to intensive care unit (ICU) after OHCA are discharged without significant neurological sequelae [1, 2].

Great efforts have been made to develop tools to identify the patients who will most benefit from intensive care [3]. Several scores, biomarkers, and/or neurological exams may help clinicians to avoid futile care by predicting poor outcomes early after ICU admission [3]. However, the use of costly and time-consuming invasive treatments may have to be considered for some patients with dismal prognosis in order to create opportunities for organs donation. This is illustrated by several cohorts that indicate that brain death (BD) may occur in a sixth to a half of successfully resuscitated patients [4–9]. Moreover, it is now established that the quality of organ from OHCA brain-dead donors is similar to that of brain-dead patients from other causes [5, 9, 10].

Predicting the occurrence of BD in comatose survivors of OHCA remains an unsolved challenge. Nevertheless, it would be useful to prognosticate BD after ICU admission. To date, only one study sought to do so but failed to determine risk factors for BD after OHCA among clinical and biological variables available at the time of ICU admission [5]. We hypothesized that data available at 24 h after ICU admission from patients alive at this point in time would be more likely to identify risk factors for BD.

## Methods

### Study design

We analyzed all consecutive OHCA patients admitted to a 15-bed university-affiliated medical ICU from 2005 to 2015. The study received approval from the local ethics committee (*Comité de Protection des Personnes Sud*-*Est II*). This institutional review board waived the need for consent given the retrospective and non-interventional design of the project.

### Data collection

During the study period, all patients aged over 18 years admitted to the ICU after a non-traumatic OHCA and alive at 24 h after ICU admission (Day 1) were included. Data regarding OHCA and cardiopulmonary resuscitation were characterized according to Utstein Style [11]. Given the possible association with a high risk of BD [7, 12, 13], the number of patients with neurological cause of cardiac arrest (stroke and status epilepticus) or hanging was also reported. The following information was also recorded at Day 1: hemodynamics and use of vasoactive drugs, temperature and targeted temperature management (TTM), continuous sedation, neurological evaluation, laboratory test data (most pejorative values during the first 24 h in ICU), Sequential Organ Failure Assessment (SOFA) score [14], and Simplified Acute Physiology Score II (SAPS II) [15].

Survival and neurological outcomes (Glasgow–Pittsburgh Cerebral Performance Categories, CPC [11]) were assessed at Day 90. Good neurological outcome was defined as a CPC 1 or 2. BD was defined according to international guidelines [16]. As recommended, the clinical diagnosis of BD was made in the absence of confounding factors (e.g., residual sedation) [16]. Ancillary tests (e.g., computed tomography angiograph or electroencephalogram) were used to confirm BD when the apnea test could not be performed and/or, according to French law, when organ donation was considered [17].

In case of BD, data collected referred to the occurrence of organ procurement, the presence of a contraindication to organ donation, refusal, or other reasons for non-procurement.

### Post-cardiac arrest care

During the study period, all patients admitted to our ICU after OHCA were managed according to international guidelines for post-cardiac arrest care [18, 19]. TTM was considered for all patients, for a period of 12–24 h, with a target temperature of 32–34 °C (external cooling). Midazolam and sufentanil were given as a continuous sedation. Sedation and analgesia protocols were based on the Richmond Agitation-Sedation Scale (RASS) and the behavioral pain scale, respectively [20]. Neuromuscular blocking agents could be used to prevent shivering during TTM. As recommended, we aimed to maintain the essential biological variables within physiological limits. There was no significant change in our protocol for post-cardiac arrest care during the study period.

### Statistical analysis

Values are presented as median and interquartile range (IQR) or number and percentage (%), as appropriate. Univariate comparisons were performed using Mann–Whitney U test for continuous variables, and Chi-square or Fisher’s exact test for categorical variables, as appropriate.

Backward stepwise multivariate analysis using a logistic regression model was performed to assess the factors predicting evolvement toward BD. Following univariate analysis, variables with *p* ≤ 0.10 and/or clinical pertinent variables were included in the model. Given uncertainty about the duration of no flow in patients with unwitnessed OHCA, these data were not entered in the model. Because of nonlinearity, the “low-flow period” data were transformed into a dummy variable according to its median value. Data which were part of the definition of BD were not included in the model. Because of the collinearity with SAPS II and/or the use of vasoactive drugs, SOFA score, epinephrine doses, mean arterial pressure, heart rate, and biological variables linked with organ dysfunctions were also not included in the model as stand-alone variables. Potential confounding factors were eliminated if *p* value was > 0.10. Odds ratios (OR) were estimated with 95% confidence intervals (95% CI). Discrimination of the model was evaluated by measuring the area under the receiver operating characteristics (ROC) curve. MedCalc Statistical Software version 12.1.2 for windows (MedCalc Software BVBA, Ostend, Belgium) was used for all analyses. The significance level was set at *p* < 0.05.

## Results

**Fig. 1:**
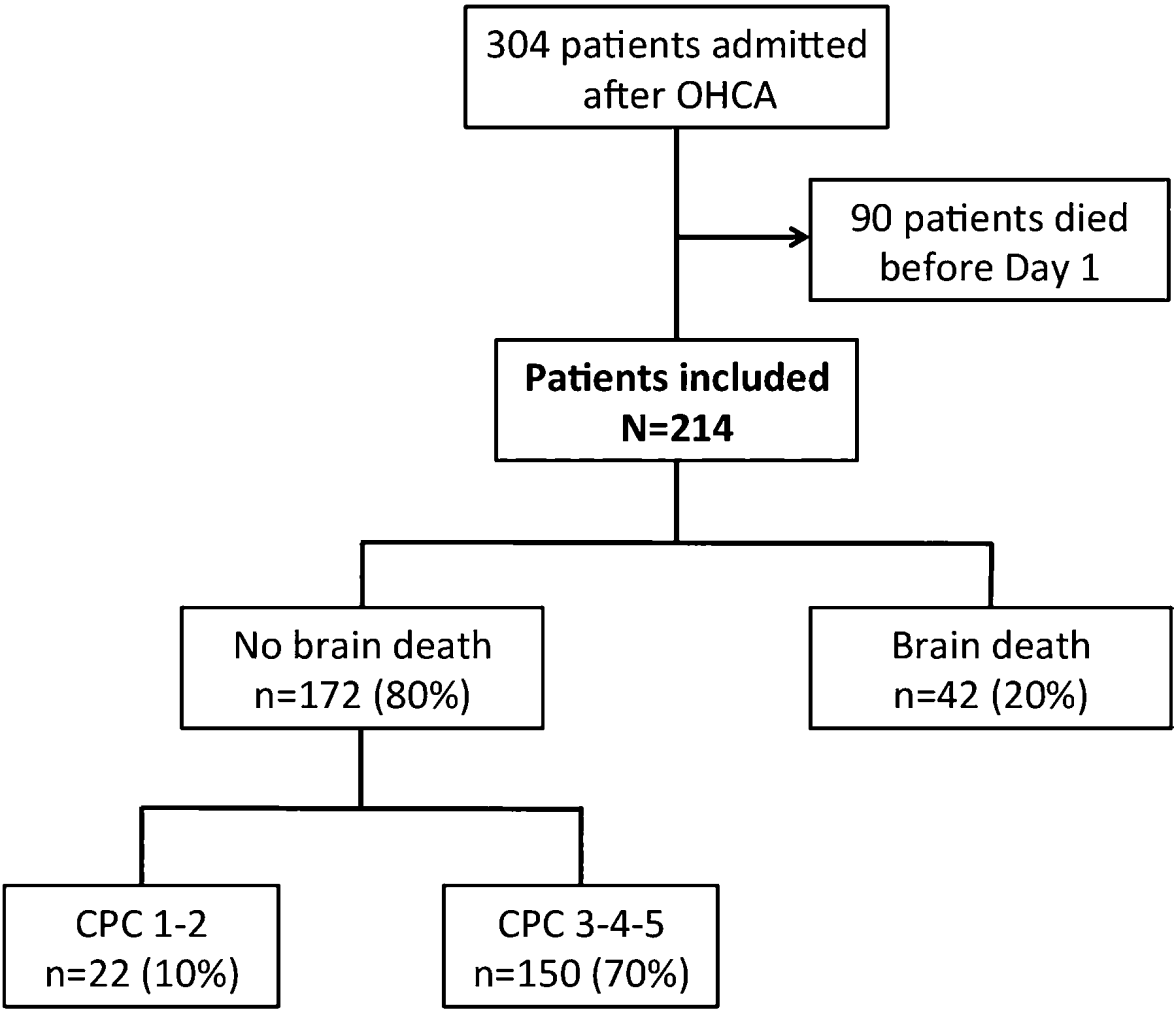
Flowchart. *OHCA* out-of-hospital cardiac arrest, *CPC* Cerebral Performance Categories

During the study period, there were 304 patients admitted to ICU after OHCA; 214 of whom were alive at Day 1 and were included in the study (Fig. 1). Among these, 22 (10%) had good neurological outcomes, whereas 42 (20%) evolved toward BD (Fig. 1). Clinical BD diagnosis was confirmed by an ancillary test in the vast majority of cases (n = 36/42, 86%). The median time elapsed between ICU admission and BD diagnosis was 2 (2–3) days. A total of 23 organs procured from 7 brain-dead patients were successfully transplanted (3 hearts, 6 livers, and 14 kidneys). In the other 35 brain-dead patients, the main reasons for non-procurement were next-of-kin refusal (n = 14) and contraindication related to multiple organ failure (n = 11).

The baseline characteristics of the patients according to BD are presented in Table 1. The majority of patients presented with OHCA of non-cardiac origin and non-shockable rhythms (Table 1). The median length of stay in ICU was 4 (2–6) days. In the no BD group, a high proportion of deaths occurred after withdrawal of life-sustaining therapy (n = 102/136, 75%) with a median delay from ICU admission of 5 (4–7) days. The age, the proportion of shockable rhythm, the proportion of witnessed OHCA, as well as resuscitation data significantly differed between patients with and without BD (Table 1). Patients with BD had a neurological cause of OHCA in 7 (17%) cases (stroke n = 5, and status epilepticus n = 2) as compared to 5/172 (3%) in the group with no BD (stroke n = 4 and status epilepticus n = 1) (*p* < 0.01). BD occurred in 7/15 (47%) patients following hanging. As shown in Table 2, patients who evolved toward BD received more often vasoactive drugs for hemodynamic instability, had more often signs of brainstem dysfunction, and had more organ dysfunction at Day 1. In contrast, the occurrence of BD was significantly less frequent when patients still had pupillary light reflex or displayed myoclonus at Day 1 (Table 2).

**Table 1:** Patient and cardiac arrest characteristics

	All patients (n = 214)	No brain death (n = 172)	Brain death (n = 42)	*p*
Age, years	68 (57–77)	70 (60–78)	58 (45–70)	< 0.001
Male gender	119 (56)	101 (59)	18 (43)	0.08
Comorbidities				
Hypertension	85 (40)	70 (41)	15 (36)	0.60
COPD	58 (27)	45 (26)	13 (31)	0.56
Congestive heart failure	33 (15)	27 (16)	6 (14)	> 0.99
Diabetes	48 (22)	43 (25)	5 (12)	0.09
Chronic renal failure	12 (6)	10 (6)	2 (5)	0.17
Presumed cause of cardiac arrest				0.30
Cardiac	44 (21)	39 (23)	5 (12)	
Non-cardiac	160 (75)	125 (73)	35 (83)	
Undetermined	10 (5)	8 (4)	2 (5)	
Location of cardiac arrest				0.48
Home	165 (77)	130 (76)	35 (83)	–
Public place	45 (21)	39 (23)	6 (14)	–
Other	4 (2)	3 (2)	1 (2)	–
Witnessed cardiac arrest	166 (78)	139 (81)	27 (64)	0.02
First recorded cardiac rhythm				0.03
Shockable	25 (12)	24 (14)	1 (2)	–
Non-shockable	189 (88)	148 (86)	41 (98)	–
Resuscitation				
Bystander CPR	90 (42)	79 (46)	11 (26)	0.02
Time from collapse to ROSC, min	25 (15–35)	22 (15–32)	32 (23–46)	< 0.001
No-flow period, min	6 (0–10)	5 (0–10)	10 (5–15)	< 0.001
Low-flow period, min	16 (10–26)	15 (9–25)	22 (15–34)	< 0.001
Dose of epinephrine, mg	3 (1–5)	3 (1–5)	3 (2–6)	0.02
Number of defibrillation attempts	0 (0–1)	0 (0–1)	0 (0–1)	0.76

Data are expressed as median (interquartile range) or number (%)

*COPD* chronic obstructive pulmonary disease, *OHCA* out-of-hospital cardiac arrest, *CPR* cardiopulmonary resuscitation, *ROSC* restoration of spontaneous circulation

**Table 2:** Clinical and biological data at Day 1

	All patients (n = 214)	No brain death (n = 172)	Brain death (n = 42)	*p*
Hemodynamics				
MAP, mmHg	78 (65–91)	80 (65–92)	73 (63–82)	0.09
Heart rate, bpm	100 (85–114)	98 (82–112)	110 (95–119)	< 0.01
Vasoactive drugs	107 (50)	74 (43)	33 (79)	< 0.01
Temperature				
Temperature, °C	37.4 (36.2–38.2)	37.5 (36.4–38.1)	37.5 (36.1–38.7)	0.80
TTM	102 (48)	81 (47)	21 (50)	0.86
Continuous sedation	88 (41)	75 (44)	13 (31)	0.16
Neurological examination				
GCS score	3 (3–4)	3 (3–5)	3 (3–3)	< 0.001
GCS Motor subscore ≤ 2	181 (85)	139 (81)	42 (100)	< 0.001
Myoclonus	68 (32)	67 (39)	1 (2)	< 0.001
Pupillary light reflex present	112 (52)	110 (64)	2 (5)	< 0.001
Spontaneous ventilation absent	75 (35)	45 (26)	30 (71)	< 0.001
Biological variables				
Arterial pH	7.25 (7.14–7.32)	7.25 (7.15–7.33)	7.17 (7.07–7.29)	0.02
PaCO_2_, KPa	5.3 (4.5–6.5)	5.3 (4.6–6.5)	5.4 (4.4–7.0)	0.97
Serum lactate, mmol/l	7.7 (4.9–11.0)	7.4 (4.5–10.7)	8.9 (5.8–12.0)	0.07
Troponin, µg/l	0.7 (0.1–5.1)	0.5 (0.1–3.6)	1,9 (0.3–19.0)	< 0.01
ALT, IU/L	104 (44–261)	84 (39–206)	178 (92–376)	< 0.001
Creatinine, µmol/l	110 (81–173)	107 (80–172)	124 (87–178)	0.33
Platelets, 10^9^/l	201 (155–256)	208 (157–261)	181 (144–242)	0.29
SOFA score	9 (6–12)	9 (6–11)	11 (9–14)	< 0.001
SAPS II	74 (65–86)	75 (64–86)	74 (69–86)	0.91

Data are expressed as median (interquartile range) or number (%)

*MAP* mean arterial pressure, *TTM* targeted temperature management, *GCS* Glasgow Coma Scale, *PaCO*_*2*_ arterial partial pressure of carbon dioxide, *ALT* alanine aminotransferase, *SOFA* Sequential Organ Failure Assessment, *SAPS II* Simplified Acute Physiology Score II

Multivariate analysis identified 5 risk factors available at Day 1, independently associated with BD after OHCA (Table 3). Neurological cause of cardiac arrest was the strongest predictor of BD, followed by the need for vasoactive drugs at Day 1 (Table 3). The area under the ROC curve was 0.84 (95% CI: 0.78–0.89).

**Table 3:** Independent predictors of brain death

	Odds ratio	95% CI	*p*
Age, per year	0.95	0.92–0.98	< 0.001
Female gender	2.34	1.02–5.35	0.043
Neurological cause of cardiac arrest	14.72	3.03–71.37	< 0.001
Duration of the low-flow period > 16 min	2.94	1.21–7.16	0.017
Vasoactive drugs at Day 1	6.20	2.41–15.93	< 0.001

*CI* confidence intervals, *ROSC* return of spontaneous circulation

## Discussion

The present study provides the first insight into risk factors for developing BD after OHCA. Female gender, young age, neurological cause of cardiac arrest, duration of the low-flow period, and persistent hemodynamic shock were independently associated with the occurrence of BD. These five simple factors might help clinicians to identify early a potential pool of future organ donors among OHCA patients.

In the present study, one in five patients who survived more than 24 h evolved toward BD. Moreover, 5 patients evolved to BD before Day 1 (data not shown). The high rate of BD following cardiac arrest has been known since the beginning of the 2000s [21]. The most recent systematic review including 23,388 patients indicates that BD might occur in about 10% of the OHCA [8]. However, this figure is probably underestimated as some patients with very poor prognosis might have died before BD diagnosis, due to intractable hemodynamic shock or early withdrawal of life support. Furthermore, one can reasonably assume that OHCA patients with the worst prognosis are often neither referred to centers of expertise nor enrolled in a study, which probably decreases their chance of being both diagnosed with BD and included in a cohort. In the aforementioned review, the authors also reported a wide range of BD incidence rates (from 0 to 43%) suggesting that this event depends on the population studied. In the present study an overwhelming majority of patients had non-shockable OHCA, which are traditionally associated with dismal outcomes [1, 2, 22, 23]. As a result, the patients were twice more likely to develop BD than to survive without severe neurological sequelae. Thus, given the high proportion of BD after OHCA, when organ donation is an option, aggressive treatment appears to be appropriate. In this context, it would be helpful to have tools to prognosticate the occurrence of BD, especially in patients with the poorest prognosis, in order to prevent futile care.

We identified five factors available at Day 1 independently associated with progression toward BD after OHCA. To the best of our knowledge, only one previous study, in which 16% of patients met criteria for BD, sought to do so [5]. Unfortunately, analyzing only data available at ICU admission, the authors failed to find independent early predictors of BD. Consequently, we chose not to include patients who died within the first 24 h after ICU admission. Furthermore, BD prognostication is probably not relevant at a very early stage as most of deaths occurring within few hours after hospital admission result from intractable cardiovascular failure and multiple organ failure, contraindicating any organ procurement [14, 24]. Moreover, because of confounding factors such as sedation or hypothermia, BD diagnosis is barely feasible soon after admission [16]. Taking into accounts these considerations, we found that female gender, young age, neurological cause of cardiac arrest, duration of the low-flow period, and persistent hemodynamic shock, were independently associated with BD after OHCA. While one can reasonably assume that cerebral edema was more likely to induce BD in patients without brain atrophy (i.e., in young patients), the reasons why females had higher chance of BD remain to be determined. The other risk factors were related to the extent of the initial brain insult or the severity of the post-cardiac arrest syndrome.

These risk factors for BD can form the basis for a future simple score system. Providing a probability of BD after OHCA, such a score might help to adjust levels of care, especially when there is no chance of neurological recovery. The prospect of organ donation may also re-motivate the teams to optimize organ protection even in patients with the worse prognosis. Equally important, identifying patients at high risk of BD is primordial for information of families/relatives, notably regarding organ donation. Besides, given the fact that the delay between ICU admission and BD is relatively short after OHCA, physicians might also opt for short-acting sedation drugs and choose a target temperature that does not alter neuro-electrophysiologic tests.

We chose not to introduce neurological exam variables at Day 1 into the logistic regression model (i.e., GCS, pupillary reactivity, or myoclonus) because of redundancy with BD diagnosis criteria [16]. However, interestingly, some of these variables were strongly associated with the absence of subsequent occurrence of BD. For instance, none of the patients with a Glasgow motor subscore > 2 at Day 1 developed BD. In the same way, patients with myoclonus and/or preserved pupillary light reflex at Day 1, were very unlikely (up to 5%) to progress to BD. Taken together, these very simple clinical variables could contribute to identify at an early stage the patients who will not meet BD criteria after OHCA.

We acknowledge some limitations. First, given the retrospective analysis of a prospective basis, we might have omitted some confounders for the adjustment. Second, we did not analyze the predictive value of some prognostic factors such as early electroencephalogram or biomarkers of brain injury. Third, even though the discrimination of the prediction model was good, we cannot rule out the possibility of an overfitting related to the relatively low number of events per variable. Finally, our study only represents the characteristics of a single hospital and we performed statistical analysis with data from patients admitted mostly after non-shockable OHCA of non-cardiac origin. Therefore, even though BD was not independently associated with initial cardiac rhythm, further studies are needed to confirm our findings, especially in cohorts where OHCAs from cardiac origin predominate.

## Conclusions

To the best of our knowledge, this study is the first to highlight demographic, anamnestic, and clinical variables independently associated with the occurrence of BD after OHCA. These findings might help physicians in their decision-making processes and could serve as a basis for developing a simple score that could accurately predict BD after OHCA.

## Abbreviations

BD: brain death
CPC: Cerebral Performance Categories
CI: confidence intervals
ICU: intensive care unit
OHCA: out-of-hospital cardiac arrest
OR: odds ratio
SOFA: Sequential Organ Failure Assessment
